# The associations between low-level gaming, high-level gaming and problematic alcohol use

**DOI:** 10.1016/j.abrep.2019.100186

**Published:** 2019-05-06

**Authors:** Eilin K. Erevik, Torbjørn Torsheim, Cecilie S. Andreassen, Elfrid Krossbakken, Øystein Vedaa, Ståle Pallesen

**Affiliations:** a Department of Psychosocial Science, University of Bergen, PO Box 7807, 5020 Bergen, Norway; b Department of Social Studies, University of Stavanger, PO Box 8600, Forus, 4036 Stavanger, Norway; c Department of Clinical Psychology, University of Bergen, PO Box 7807, 5020 Bergen, Norway; d Department of Health Promotion, Norwegian Institute of Public Health, PO Box 973, Sentrum, 5808 Bergen, Norway; e Department of Mental Health, Norwegian University of Science and Technology, PO Box 8905, NO-7491 Trondheim, Norway

**Keywords:** Gaming, Gaming disorder, Alcohol use, Students, Personality, Mental health

## Abstract

The current study aimed to investigate associations between gaming and different patterns of problematic alcohol use, controlling for important demographics, personality and mental health covariates. Data was collected by an online survey during fall 2016 (*N* = 5217). Students who had participated in a survey among students in Bergen, Norway, one year earlier were invited to participate. Crude and adjusted binary logistic regression analyses were conducted in order to assess the relationship between different patterns of problematic alcohol use and gaming (i.e. low-level gaming and high-level gaming vs. no gaming) while controlling for important covariates. The different gaming groups were categorised based on the number of symptoms of “gaming addiction” (in total seven) that they endorsed: 4 > symptoms = low-level gaming, 4 ≤ symptoms = high-level gaming. Only 0.2% (*n* = 11) endorsed all seven symptoms. Low-level gaming was positively associated with patterns of problematic alcohol use in the crude analyses; these associations became non-significant when controlling for demographic variables. High-level gaming was inversely associated with patterns of problematic alcohol use when controlling for demographics, personality, and mental health covariates. The inverse relationship between high-level gaming and problematic alcohol use (when controlling for covariates) suggest that heavy investment in gaming may protect against excessive alcohol use and alcohol-related harm. Possible explanations discussed for the inverse associations include high-level gamers having less available time to drink, intoxication being incompatible with gaming, and/or high-level gamers experiencing sufficient satisfaction/escape and social bonding by gaming, hence having less need for alcohol.

## Highlights

• Low-level gaming was associated with problem drinking in the crude analyses.
• This association was explained low-level gamers' demographical characteristics.
• High-level gaming was contrary linked to problem drinking in the adjusted analyses.
• High-level gaming may protect against alcohol use and alcohol-related harm.

## 1 Introduction

Alcohol use and playing video games are common recreational activities among young adults and students (Erevik, Pallesen, Vedaa, Andreassen, & Torsheim, 2017; Ipsos MediaCT, 2014; Mentzoni et al., 2011; World Health Organization, 2014). Both alcohol and video games are used to enhance positive feelings and suppress negative ones, and both can play an important part in social bonding (Cooper, Frone, Russell, & Mudar, 1995; Griffiths, 2010; Hellström, Nilsson, Leppert, & Åslund, 2012; Hoffman & Nadelson, 2010; Sayette et al., 2012). Alcohol in particular, however, can also involve a great deal of harm for the user and has clear and well-documented addictive properties (Rehm et al., 2003; Vengeliene, Bilbao, Molander, & Spanagel, 2008). In the case of alcohol misuse, potential harms have been documented such as accidents, organ damage, and cancer (Rehm et al., 2003). Consumption of many alcohol units on a single occasion is oftentimes termed binge drinking, and such a pattern of alcohol use is common among young adults/students and potentially harmful (Bingham, Shope, & Tang, 2005; Slutske et al., 2004; Wechsler, Dowdall, Davenport, & Castillo, 1995). There is disagreement regarding the alcohol quantity that constitutes binge drinking. Drinking 5–6 alcohol units or more on the same occasion is a frequently used cut-off for binge drinking (Connor, Gray, & Kypri, 2010; Wechsler et al., 1995), but several authors have argued that this threshold could be too low (Jackson, 2008; Read, Beattie, Chamberlain, & Merrill, 2008). Read et al. (2008) suggest that cut-offs of 6 and 7 alcohol units (for women and men, respectively) discriminate college students at greater risk of reaching intoxication and experiencing adverse alcohol consequences fairly well.

Heavy investment in gaming has also been suggested to involve harm for some individuals (Kuss & Griffiths, 2012; Liu & Peng, 2009). The harms associated with heavy investment in video games seem, however, not as detrimental or irreversible as those alcohol users can experience. Potential harms associated with gaming includes disturbance of other areas of life (e.g. school/work and health behaviours), which can be regarded as a sign of “addiction”, other addiction-like symptoms (e.g. loss of control), mental health problems (but this is not consistently found), and loneliness (Krossbakken et al., 2018; Kuss & Griffiths, 2012; Liu & Peng, 2009; Przybylski, Weinstein, & Murayama, 2016). Several initiatives to identify those at risk of gaming-related harm have been conducted; most of these initiatives are based on traditional conceptualisations of addiction where symptoms include salience, tolerance, mood modification, relapse, withdrawal, conflict and problems, such “addiction” symptoms are also considered as potential harms related to gaming in themselves (Lemmens, Valkenburg, & Peter, 2009). Based on the studies demonstrating that some gamers experience addictionlike symptoms related to video games and that this can be associated with mental distress, “Gaming Disorder” and “Internet Gaming Disorder” have been included as a diagnosis and as a tentative diagnosis in the 11th version of the International Classification of Diseases (ICD-11) and in the 5th edition of the Diagnostic and Statistical Manual of Mental Disorders (DSM-5), respectively (American Psychiatric Association, 2013; World Health Organization, 2018). The criteria for gaming disorder/internet gaming disorder are based on conceptualisations of traditional addiction symptoms. Both the use of addiction terminology related to gaming and the gaming disorder diagnoses are, however, controversial and critics have argued that the scientific basis and utility of a gaming disorder diagnosis is not sufficiently established (Van Rooij et al., 2018). It has been reasoned that addictionlike symptoms related to behaviours may reflect investment rather than addiction (Aarseth et al., 2017; Carras & Kardefelt-Winther, 2018). For instance, the symptom of salience, i.e. spending a lot of time thinking about the object of addiction, may not reflect a problem in the context of gaming, as spending quite a bit of time planning, calculating and discussing the next game can be a normal, unproblematic way of optimizing the gaming experience (Carras & Kardefelt-Winther, 2018). Accordingly, it has been argued that the current diagnostic conceptualisations of gaming disorders have a poor specificity and that the group who qualifies for the diagnoses may also consist of individuals who are invested in but not harmed by their gaming (Aarseth et al., 2017; Carras & Kardefelt-Winther, 2018). The claim of the diagnoses as having too poor specificity is supported by one study which suggested that the conceptualization of gaming disorder in DSM-5 may lead to erroneous classifications of around one third of gamers (Carras & Kardefelt-Winther, 2018). Further, the directionality between gaming and associated distress seems somewhat unclear (Van Rooij et al., 2018). Given the controversy surrounding the nature of problems associated with gaming and the gaming disorder diagnosis, investigating the relationship between gaming and difficulties like alcohol problems, seems particularly important and timely.

### 1.1 Theoretical assumptions on the relationship between gaming and alcohol use

Several claims have been made as to how different potentially addictive behaviours, such as alcohol use and gaming, may relate to each other. For one, it has been suggested that different addictions are likely to co-occur, as different addictions appear to have similar predictors (e.g. impulsivity) (Choi et al., 2014; Jacobs, 1986; Walther, Morgenstern, & Hanewinkel, 2012). According to replacement theory on the other hand, different addictive substances and behaviours, may replace each other (Sussman & Black, 2008). A third perspective is that addiction of one substance or behaviour increases the likelihood of other addictions, as addiction in general decreases one's sensitivity for dopamine and weakens own control, which may make the individual more vulnerable for additional addictions (Ream, Elliott, & Dunlap, 2011). These three perspectives on how gaming may relate to alcohol use may also apply if one views gaming exclusively as a leisure activity and not as a potential addiction. Several leisure activities (e.g. exercise) may have some functional similarities with alcohol use (e.g. stimulating dopamine release, satisfying important needs such as social cohesion) (Dishman et al., 2006; Kilpatrick, Hebert, & Bartholomew, 2005). Hence, one can still reason that gaming and alcohol use may either co-occur, replace each other, or that one may enhance the use of the other.

Many of the same factors seem to predict both alcohol use and gaming. Overlapping predictors of gaming and alcohol use suggest that the two behaviours may co-occur, as those who seek out alcohol may also be the ones who engage in gaming. Identifying overlapping predictors is important in order to understand the relationship between alcohol use and gaming. Demographical characteristics, personality traits, and mental health factors appear to be important antecedents of both alcohol use and gaming (Erevik et al., 2017; Kuss & Griffiths, 2012; Wittek et al., 2016). Increasing alcohol use and increasing gaming investment and/or problems associated with gaming have both been found to be predicted by male sex, younger age, being single, lower scores on agreeableness and conscientiousness, higher scores on neuroticism, and symptoms of depression and anxiety (Andersson, Johnsson, Berglund, & Ojehagen, 2007; Dixit & Crum, 2000; Erevik et al., 2017; Ferguson, Coulson, & Barnett, 2011; Grant et al., 2004; Kushner, Abrams, & Borchardt, 2000; Kuss & Griffiths, 2012; Malouff, Thorsteinsson, Rooke, & Schutte, 2007; Mentzoni et al., 2011; Peters & Malesky, 2008; Van Rooij et al., 2014; Wenzel, Bakken, Johansson, Götestam, & Øren, 2009; Wittek et al., 2016).

According to the replacement theory, excessive alcohol use and heavy investment in gaming may replace each other, i.e. be inversely causally associated (Sussman & Black, 2008). Replacement theory emphasizes that an addiction serves key functions (e.g., relaxation, escape, excitement, social lubrication) and desensitizes the reward system. Cessation of an addiction may thus make the individual vulnerable for additional addictions in the search of satisfying needs and obtaining reward (Sussman & Black, 2008). According to the replacement theory, the addicted individual may seek out substitute addictions that are less harmful than the original (Sussman & Black, 2008). Given that excessive alcohol use is more harmful than heavy investment in gaming, one would therefore expect gaming to replace alcohol use and not vice versa. The replacement theory focuses on addiction and substitute addiction. Other routes through which gaming may reduce or prevent excessive alcohol use are also conceivable. Gaming, in line with a range of other activities commonly not regarded as addictive (e.g. exercise), can provide positive experiences that may protect against excessive alcohol use (e.g. achievement, good self-esteem, and social support) and as such serve some of the same functions as alcohol without being a substitute addiction per se (Bergen, Martin, Roeger, & Allison, 2005; DeSimone, Murray, & Lester, 1994; Griffiths, 2010; Kilpatrick et al., 2005; Menagi, Harrell, & June, 2008). Gaming may also reduce alcohol use simply by taking up time, leaving the individual less time to drink. A final route through which gaming may reduce alcohol use is through depending on and enhancing cognitive function, in particular executive function (i.e. higher order cognitive abilities, including attentional control, planning, inhibition) (Giancola, Zeichner, Yarnell, & Dickson, 1996). Performance in video games often relies on executive functions (Boot, Kramer, Simons, Fabiani, & Gratton, 2008) which are impaired under alcohol influence (Peterson, Rothfleisch, Zelazo, & Pihl, 1990; Wiese, Shlipak, & Browner, 2000), and gamers may thus avoid alcohol use while gaming in order to perform better in the video games. Further, some studies have suggested that gaming may improve executive function (Andrews & Murphy, 2006; Boot et al., 2008; Buelow, Okdie, & Cooper, 2015). Impaired executive function is further believed to be an important determinant of excessive alcohol use (Giancola et al., 1996). Hence, gaming's potential enhancing effects on executive function may protect against excessive alcohol use.

In some instances, different addictions clearly co-occur (Sussman & Black, 2008), and it has been speculated that having one addiction may increase the likelihood of additional addictions (Ream et al., 2011). From this perspective, one would expect heavy investment in gaming to increase the likelihood of excessive alcohol use, and vice versa. Pathways through which one addiction may increase the risk of additional addictions include the reduced self-control, decreased dopamine sensitivity, and increased psychological distress that accompanies substance addictions and possibly behavioral addictions as well (Baler & Volkow, 2006; Boden & Fergusson, 2011; Karim & Chaudhri, 2012). Reduced self-control, decreased dopamine sensitivity, and increased psychological distress may further make the individual more susceptible to additional addictions. One could still reason that gaming may increase alcohol use, and vice versa, if gaming is viewed as a leisure activity rather than an addiction. Both gaming and alcohol use are often social activities (Griffiths, 2010; Park, 2004), and engaging in one of them may increase the other through expanding the individual's social network (e.g. meeting new friends through gaming and going to parties with the new friends). The claim that leisure activities may increase alcohol use (perhaps via expansion of the social network) is supported by findings suggesting that engagement in sports may increase alcohol use (Lisha & Sussman, 2010).

### 1.2 Empirical studies on the relationship between gaming and alcohol use

Studies on the association between gaming and alcohol use have generally either found a positive association between gaming and alcohol use, or have found no association (Brunborg, Mentzoni, & Frøyland, 2014; Krossbakken et al., 2018; Padilla-Walker, Nelson, Carroll, & Jensen, 2010; Van Rooij et al., 2014; Wenzel et al., 2009). The existing studies on alcohol use and gaming entail, however, some limitations. For one, gaming may relate discordantly to different patterns of alcohol use. No studies have so far looked into how gaming is associated with different types of drinking patterns. Further, the studies investigating the association between gaming and alcohol use have not included the wide range of covariates that may explain both gaming and alcohol use. Including and identifying covariates that may act as “third variables” in the relationship between gaming and alcohol use is important, as such knowledge may expand our understanding of the causal mechanisms behind the relationship between gaming and alcohol use.

### 1.3 Study objectives

The current study aimed to investigate the association between lower and higher levels of problems associated with gaming/gaming investment and problematic alcohol use in a large sample consisting of students/former students. A second aim was to investigate if demographic, personality, or mental health might act as confounders in the relationship between gaming and problematic alcohol use.

## 2 Methods

### 2.1 Procedures and sample

Data was collected by an online survey during the fall of 2016. Individuals who participated in a survey among students in Bergen, Norway, during fall 2015 (response rate: 39.4%) were invited to participate in a follow-up survey including questions on gaming. The students who were invited to participate in the 2015 survey were from all study levels (i.e. both undergraduate and graduate studies, although PhD-students were not invited to participate). A total of 5217 individuals agreed to participate in the 2016 survey (response rate: 51.5%), and these participants comprised the sample in the current study. It should be noted that by fall 2016, a substantial proportion of the sample were no longer students (approximately 40% of students in Bergen ends their education yearly) and might not have access to their university e-mail account. Consequently, the response rate for those who actually received the invitation was probably higher than 51.5%. The sample had a mean age of 25.8 years, 64.8% were women, 92.7% were born in Norway, and 83.7% were still students at the time of the 2016 survey. Participants were given information about the study, data-storage and use, potential risk and benefits associated with participation, and their right to abstain from participation, before they could choose to respond to the survey. The Regional Committee for Medical and Health Research Ethics, health region western Norway (project number 2015/1154) approved the study.

### 2.2 Measurements

#### 2.2.1 Gaming

The participants were first asked if they had played any video games (on computers, PlayStation, PSP, Nintendo, Xbox, Gameboy, mobile phones or similar) the last six months. Those who reported having played video games were further administered the 7-item Gaming Addiction Scale for Adolescents (GASA; Lemmens et al., 2009). The GASA measures the extent to which respondents have experienced seven symptoms of “video game addiction” (i.e. salience, tolerance, mood modification, relapse, withdrawal, conflict, and problems) the last six months (response options [range, 1–5]: never; rarely; sometimes; often; very often). According to Lemmens et al. (2009), endorsement of at least four of the items of the GASA (i.e. reporting experiencing a symptom “sometimes” or more often) indicates that the respondent may have a problematic relationship to video games. Endorsement of all seven items is often considered as an indication of pathological gaming/video game addiction (Lemmens et al., 2009; Mentzoni et al., 2011). Given the controversy regarding the nature of problems associated with gaming and gaming addiction, we regarded endorsement of more items of GASA to indicate higher levels of problems associated with gaming and higher levels of gaming investment, but refrained from categorizing the respondents as problem gamers, pathological gamers, addicted gamers, and the like. In the current study, the items of the GASA obtained a Cronbach's alpha of 0.80.

#### 2.2.2 Alcohol use

Alcohol use was assessed by the 10-item Alcohol Use Disorders Identification Test (AUDIT; Babor, Higgins-Biddle, Saunders, & Monteiro, 2001; Bohn, Babor, & Kranzler, 1995). The AUDIT measures different aspects of alcohol consumption, alcohol harm, and alcohol dependency symptoms the last year. The response options for the AUDIT items vary somewhat [range, 0–4]. For the majority of items, the respondents are asked to indicate how often they have experienced the specific type of alcohol harm, dependency symptom, or consumed alcohol in a particular way (i.e. never; less than monthly; monthly; weekly; daily or almost daily). Composite scores are computed ranging between 0 and 40, where scores at or above 8, 16, and 20 are considered as an indication of hazardous, harmful, and dependent alcohol use, respectively (Babor et al., 2001). In the current study, the items of the AUDIT had an acceptable internal reliability with a Cronbach's alpha of 0.79.

#### 2.2.3 Demographics

The participants were asked to report their age, sex (man; woman), if they had children (yes; no), relationship status (i.e. single; steady romantic partner, but living alone; in a cohabitant relationship; married/registered partnership; other), country of birth (Norway; Nordic country outside of Norway; European country outside of the Nordic countries; Asia, Africa, Central or South America; North America, Oceania; I don't know), and whether they were still students at the time of the follow-up survey (yes; no). Some of the demographic items were identical to the ones used in a previous study on student mental health and welfare (Nedregård & Olsen, 2014).

#### 2.2.4 Personality

We measured personality with the 20-item Mini-International Personality Item Pool (Mini-IPIP; Donnellan, Oswald, Baird, & Lucas, 2006). The Mini-IPIP assesses the five-factor model's personality traits, which are extroversion, agreeableness, conscientiousness, neuroticism, and openness (the authors of Mini-IPIP labels openness as intellect/imagination). The respondents are asked to indicate to which degree statements concerning typical behaviour describes them (response options [range, 1–5]: very wrong; somewhat wrong; neither wrong nor right; somewhat right; very right). There are four items for each of the five personality traits, and for each trait, total score ranges from 4 to 20. In the current study, the items measuring extroversion, agreeableness, conscientiousness, neuroticism, and intellect/imagination had Cronbach's alphas of 0.83, 0.79, 0.69, 0.76, and 0.75, respectively.

#### 2.2.5 Mental health

Mental health was measured with the 25-item Hopkins Symptoms Checklist (HSCL-25; Derogatis, Lipman, Rickels, Uhlenhuth, & Covi, 1974). The HSCL-25 assesses symptoms of anxiety and depression, where the respondents are asked to rate the level of symptoms experienced the past two weeks (response options [range, 1–4]: not at all; a little; quite a bit; extremely). Total score ranges from 10 to 40 for anxiety, and from 15 to 60 for depression. In the current study, the Cronbach's alpha for the items measuring symptoms of depression was 0.90, while the Cronbach's alpha for the items measuring symptoms of anxiety was 0.82.

### 2.3 Analyses

All analyses were conducted with IBM SPSS Statistics 24. Missing data were deleted list-wise.

We started by comparing low-level and high-level gamers to non-gamers on the demographic (i.e. age, sex, parental status, relationship status, country of birth, and student status), personality (i.e. extroversion, agreeableness, conscientiousness, neuroticism, intellect/imagination), and mental health variables (i.e. depression, anxiety). Student status was included in the analyses because we assumed that gaming might relate differently to problematic alcohol use among students, compared to former students. The participants who reported no use of video games the last six months were defined as non-gamers, those who reported gaming the last six months and endorsed less than four symptoms of “video game addiction” (i.e. reporting experiencing a symptom “sometimes” or more often) were defined as low-level gamers. Those who endorsed four symptoms or more were defined as high-level gamers. We reasoned that the high-level gaming group consisted of individuals with a higher level of gaming investment and some individuals who probably experienced problems related to their gaming. Endorsing all the seven symptoms of “video game addiction” gives a stronger indication of pathological/addicted gaming, this group was collapsed into the high-level gaming group as only 0.2% (*n* = 11) of the current sample endorsed all seven symptoms. Age, extroversion, agreeableness, conscientiousness, neuroticism, intellect/imagination, symptoms of depression, and symptoms of anxiety were treated as continuous variables. The other variables, sex (woman vs. man), parental status (having children vs. not having children), relationship status (single vs. not single), country of birth (Norway vs. countries outside of Norway), and student status (student vs. non-student), were dichotomous. We tested group differences with independent sample *t*-tests and chi-square tests. The effect sizes of the group differences were reported as Cohen's *d*s or phi coefficients. Cohen's *d*s of 0.20, 0.50, and 0.80 represent small, moderate, and large effect sizes, respectively, while phi coefficients of 0.10, 0.30, and 0.50 represent small, moderate, and large effect sizes (Cohen, 1988).

Further, we conducted six sets of binary logistic regression analyses. The dependent variables were hazardous (i.e. AUDIT ≥ 8), harmful (i.e. AUDIT ≥ 16), and dependent alcohol use (i.e. AUDIT ≥ 20), frequent alcohol consumption (i.e. 2 times a month or more often) and binge drinking (having a typical alcohol consumption of 5/7 alcohol units or more on the same occasion for women and men, respectively). Read et al. (2008) suggest a cut-off of 6 alcohol units as indicative of binge drinking among women. The item measuring typical alcohol quantity consumed in the AUDIT does not include a response option for 6 alcohol units or more, and hence we chose a cut-off of 5 alcohol units for women. The independent variables were gaming (i.e. no gaming vs. low-level and high-level gaming). First, we conducted crude analyses (only gaming was included as a variable). Further, we controlled for the demographical variables, the personality variables, and the mental health variables block-wise, one block at a time. Finally, we conducted fully adjusted analyses, where both the demographic, personality, and mental health variables were included as covariates. The associations between the independent and dependent variables were reported in terms of odds ratios (*OR*s). *OR* is considered as an indication of effect size, but there are no clear guidelines on how the magnitude of *OR*s should be interpreted, as this interpretation depends on the rate of the outcome of interest (Chen, Cohen, & Chen, 2010; Durlak, 2009; Rosenthal, 1996). Ferguson (2009) suggests that *OR*s of 2.0, 3.0, and 4.0, indicates small, moderate, and large effect sizes, respectively. Ferguson (2009) emphasizes that *OR*s below 2.0 are hard to interpret in terms of effect size. Common outcomes will result in smaller *OR*s, given that problematic alcohol use is quite common in our sample (Erevik et al., 2017), it is reasonable to expect that most of the *OR*s in the current analyses will be below 2.0 and hence difficult to interpret in terms of magnitude.

## 3 Results

A total of 46.1% of the sample reported no use of video-/online games the last six months, 49.9% were in the low-level gaming group, and 4.0% were in the high-level gaming group.

The results from the comparison of low-level gamers and high-level gamers to non-gamers on the included demographic, personality and mental health variables are shown in Table 1. The low-level gamers and high-level gamers differed from the non-gaming group on several of the variables; only significant (*p* < .05) differences are reported in this section. Low-level and high-level gamers were younger, less likely to be female, and less likely to have children, compared to the non-gamers. Further, there were more students in the low-level gaming group compared to the non-gaming group. In terms of personality, low-level and high-level gamers scored lower on extroversion, agreeableness and conscientiousness, and higher on intellect/imagination, compared to non-gamers. In addition, low-level gamers scored lower on neuroticism than non-gamers. As compared to non-gamers, low-level gamers had lower scores on depression and anxiety, while high-level gamers had higher scores on these measures.

**Table 1:** Comparison of non-gamers versus low-level, and high-level gamers on demographical, personality, and mental health variables (*n* = 4942).

	Non-gamers (*n* = 2276)	Low-level gamers (*n* = 2467)	Difference low-level gamers versus non-gamers	High-level gamers (*n* = 199)	Difference high-level gamers versus non-gamers
	*M* (*SD*)/% (95% CI)	*M* (*SD*)/% (95% CI)	*Cohen's d/Phi*	*M* (*SD*)/% (95% CI)	*Cohen's d/Phi*
Demographics					
Age	26.5 (7.4)	25.3 (5.2)	Cohen's *d* = 0.202⁎⁎⁎	25.2 (4.8)	Cohen's *d* = 0.382⁎⁎
Women	82.8% (81.2–84.3%)	51.1% (49.2–53.1%)	Phi = 0.335⁎⁎⁎	27.6% (21.4–33.9%)	Phi = 0.364⁎⁎⁎
Have children	16.3% (14.8–17.9%)	10.3% (9.1–11.5%)	Phi = 0.089⁎⁎⁎	7.0% (3.5–10.6%)	Phi = 0.070⁎⁎
Single	42.8% (40.9–44.9%)	44.3% (42.3–46.2%)	Phi = 0.014	38.2% (31.4–45.0%)	Phi = 0.026
Born in Norway	92.5% (91.4–93.6%)	93.1% (92.1–94.1%)	Phi = 0.011	93.9% (90.6–97.3%)	Phi = 0.015
Student	82.4% (80.9–84.0%)	85.0% (83.6–86.4%)	Phi = 0.035⁎	85.9% (81.1–90.8%)	Phi = 0.025

Personality^a^					
Extroversion	14.3 (3.6)	13.7 (3.7)	Cohen's *d* = 0.168⁎⁎⁎	12.9 (4.0)	Cohen's *d* = 0.624⁎⁎⁎
Agreeableness	17.3 (2.5)	16.6 (2.9)	Cohen's *d* = 0.278⁎⁎⁎	15.0 (3.5)	Cohen's *d* = 1.262⁎⁎⁎
Conscientiousness	15.3 (3.1)	14.3 (3.2)	Cohen's *d* = 0.341⁎⁎⁎	12.4 (3.1)	Cohen's *d* = 0.533⁎⁎⁎
Neuroticism	11.5 (3.6)	10.7 (3.7)	Cohen's *d* = 0.239⁎⁎⁎	11.9 (3.9)	Cohen's *d* = 0.052
Intellect/imagination	14.2 (3.3)	14.9 (3.2)	Cohen's *d* = 0.225⁎⁎⁎	15.1 (3.2)	Cohen's *d* = 0.152⁎⁎⁎

Mental health					
Symptoms of depression^b^	24.6 (7.6)	24.1 (7.2)	Cohen's *d* = 0.063⁎	28.9 (8.3)	Cohen's *d* = 0.938⁎⁎⁎
Symptoms of anxiety^c^	15.3 (4.3)	15.0 (4.0)	Cohen's *d* = 0.091⁎⁎	16.7 (5.2)	Cohen's *d* = 0.476⁎⁎⁎

a Total scores range 4–20 for each trait.

b Total scores range from 15 to 60.

c Total scores range from 10 to 40.

⁎ *p* < .05.

⁎⁎ *p* < .01.

⁎⁎⁎ *p* < .001.

The results from the binary regression analyses investigating the associations between gaming and alcohol use are presented in Table 2. Compared to non-gamers, low-level gamers had a higher likelihood of having a hazardous- and harmful alcohol consumption in the crude analyses. The differences between non-gamers and low-level gamers were no longer significant when demographic variables were controlled for, and there were no differences between non-gamers and low-level gamers on any of the five aspects of problematic alcohol use in the fully adjusted analyses.

**Table 2:** The association between gaming and problematic alcohol use. Binary logistic regression analyses, *n* = 4917.

	Hazardous alcohol use (AUDIT ≥ 8)	Harmful alcohol use (AUDIT ≥ 16)	Dependent alcohol use (AUDIT ≥ 20)	Frequent drinking (2 times a month+)	Usually binge drinks (5/7 ≤ alcohol units)^a^
	*OR* (95% CI)	*OR* (95% CI)	*OR* (95% CI)	*OR* (95% CI)	*OR* (95% CI)
Crude analyses					
No gaming	1.00	1.00	1.00	1.00	1.00
Low-level gaming	1.22 (1.08–1.36)⁎⁎	1.54 (1.19–2.00)⁎⁎	1.17 (0.76–1.80)	1.11 (0.98–1.25)	0.96 (0.85–1.07)
High-level gaming	1.25 (0.94–1.68)	1.44 (0.78–2.68)	0.30 (0.04–2.18)	0.55 (0.41–0.74)⁎⁎⁎	0.73 (0.55–0.98)⁎

Adjusted for demographics^b^					
No gaming	1.00	1.00	1.00	1.00	1.00
Low-level gaming	0.91 (0.80–1.04)	1.03 (0.78–1.37)	0.74 (0.46–1.19)	0.99 (0.87–1.13)	0.98 (0.87–1.12)
High-level gaming	0.80 (0.58–1.09)	0.81 (0.43–1.55)	0.16 (0.02–1.18)	0.46 (0.34–0.63)⁎⁎⁎	0.81 (0.59–1.11)

Adjusted for personality^c^					
No gaming	1.00	1.00	1.00	1.00	1.00
Low-level gaming	1.19 (1.05–1.35)⁎⁎	1.37 (1.04–1.80)⁎	0.96 (0.61–1.51)	1.13 (0.99–1.30)	1.06 (0.94–1.19)
High-level gaming	0.97 (0.71–1.32)	0.74 (0.38–1.42)	0.12 (0.01–0.88)⁎	0.53 (0.39–0.73)⁎⁎⁎	0.76 (0.55–1.03)

Adjusted for mental health^d^					
No gaming	1.00	1.00	1.00	1.00	1.00
Low-level gaming	1.25 (1.11–1.40)⁎⁎⁎	1.57 (1.21–2.05)⁎⁎	1.20 (0.78–1.85)	1.09 (0.96–1.24)	1.02 (0.90–1.14)
High-level gaming	1.14 (0.85–1.53)	1.13 (0.60–2.12)	0.22 (0.03–1.61)	0.57 (0.43–0.77)⁎⁎⁎	0.73 (0.54–0.98)⁎

Fully adjusted^e^					
No gaming	1.00	1.00	1.00	1.00	1.00
Low-level gaming	0.91 (0.80–1.05)	1.06 (0.79–1.43)	0.77 (0.47–1.25)	1.03 (0.89–1.18)	1.03 (0.90–1.18)
High-level gaming	0.58 (0.42–0.81)⁎⁎	0.46 (0.23–0.89)⁎	0.08 (0.01–0.59)⁎	0.46 (0.33–0.64)⁎⁎⁎	0.75 (0.54–1.04)

a Binge drinking was defined as 5 or more alcohol units for women and 7 or more alcohol units for men.

b Adjusted for age, sex, parental status, relationship status, country of birth, and student status.

c Adjusted for extroversion, agreeableness, conscientiousness, neuroticism, and intellect/imagination.

d Adjusted for symptoms of depression and anxiety.

e Adjusted for demographics, personality, and mental health.

⁎ *p* < .05.

⁎⁎ *p* < .01.

⁎⁎⁎ *p* < .001.

In the crude analyses, high-level gaming was inversely associated with frequent drinking and binge drinking. The inverse association between high-level gaming and frequent drinking prevailed in the partly adjusted analyses as well as in the fully adjusted analysis. The inverse association between high-level gaming and binge drinking was not significant when demographic and personality variables were adjusted for, and there was no association between high-level gaming and binge drinking in the fully adjusted analysis. Compared to non-gamers, high-level gamers were less likely to have a hazardous or harmful alcohol consumption when demographics, personality, and mental health factors were controlled for. In addition, high-level gaming was inversely associated with dependent alcohol use both in the analysis where only personality was adjusted for and in the fully adjusted analysis.

## 4 Discussion

The current study found low-level gaming and problematic alcohol use to be positively related when not adjusting for other variables. However, these associations were no longer significant when demographic, personality, and mental health variables were adjusted for. Demographic characteristics associated with low-level gaming appeared to be the most important explanation to the associations between low-level gaming and problematic alcohol use. Compared to non-gamers, low-level gamers were younger, more likely to be male, less likely to have children, and more likely to be students; all of which are characteristics that have been associated with problematic alcohol use as well (Erevik et al., 2017; O'Malley & Johnston, 2002). Hence, the current results suggest that age, sex, parental status, and student status are likely to be important covariates in the relationship between low-level gaming and problematic alcohol use.

High-level gaming was inversely related to problematic alcohol use when demographic, personality, and mental health factors were controlled for. Most of the demographics, personality, and mental health characteristics that were associated with high-level gaming in the present study, have also been associated with problematic alcohol use in previous research (Erevik et al., 2017). As such, the present study suggests that high-level gamers may possess characteristics that make them more vulnerable to problematic alcohol use. Still, high-level gamers did not differ considerably from the non-gamers in terms of problematic alcohol use in the crude analyses, and high-level gamers were actually less likely to report problematic alcohol use compared to non-gamers, when adjusting for the aforementioned characteristics. The current results suggest as such that heavy investment in gaming may protect against excessive alcohol use and alcohol-related harm, and save some individuals, who may otherwise have been vulnerable, from serious and irreversible alcohol-related harm. If the inverse association between high-level gaming and problematic alcohol use reflects that gaming protects against problematic alcohol use, this would be in line with the replacement theory of addiction (Sussman & Black, 2008). It is important to note, however, that even if gaming may “replace” problematic alcohol use among high-level gamers; this does not necessarily imply that the high-level gamers have problems related to or an addiction of gaming. Instead of gaming being an evil replacing another evil (i.e. problematic alcohol use), gaming may rather be a positive leisure activity for high-level gamers and act as an antidote towards excessive alcohol use rather than as a substitute addiction. Several pathways through which gaming may reduce alcohol use are conceivable. For one, high-level gamers may use most of their spare time on gaming and thus have less time available to consume alcohol and to partake in arrangements involving alcohol use. Further, high-level gaming may protect against alcohol use due to the relationship between gaming and cognitive functions. High-level gamers may be motivated to avoid alcohol in order to not negatively affect their gaming feat as alcohol is known to worsen the users' performance on complex cognitive task, such as video games, both while under the influence and during the hangover period (Peterson et al., 1990; Wiese et al., 2000). In addition, gaming may improve cognitive function (including executive function) (Andrews & Murphy, 2006; Boot et al., 2008; Buelow et al., 2015), and improved executive function could further protect against excessive alcohol use (Giancola et al., 1996). Finally, it is possible that high-level gaming may protect against problematic alcohol use due to high-level gamers satisfying their need for enhancement of positive emotions, distraction from negative ones (escape), or social bonding through video games playing, and consequently having less need for alcohol. The design was cross-sectional, and consequently one cannot conclude that gaming does protect against problematic alcohol use. The inverse association between high-level gaming and problematic alcohol use may instead be explained by unmeasured third variables. Further, it is also possible that problematic alcohol use reduces the likelihood of high-level gaming and not the other way around. The claim that problematic alcohol use may decrease gaming investment and/or problems associated with gaming is supported by a recent study which found increasing alcohol consumption to inversely predict what the authors termed problem gaming (Krossbakken et al., 2018). More longitudinal studies are needed in order to understand the temporal relationship between high-level gaming and problematic alcohol use (e.g. whether an increase in alcohol use will be associated with a subsequent decrease in gaming behaviour and/or vice versa).

The current study is one of very few studies investigating the association between gaming and problematic alcohol use. Previous research on the associations between gaming and alcohol use has differed greatly in the measurements of alcohol use and gaming (Brunborg et al., 2014; Krossbakken et al., 2018; Padilla-Walker et al., 2010; Van Rooij et al., 2014; Wenzel et al., 2009). The variation in measurements in previous research precludes a clear synthetisation of their results, but in general, previous studies have reported a positive association between increasing levels of gaming investment and/or problems associated with gaming and increasing levels of alcohol use (Brunborg et al., 2014; Padilla-Walker et al., 2010; Van Rooij et al., 2014; Wenzel et al., 2009). The multifarious of covariates included in the present study may explain the discrepancy between our results and those of previous studies. Further, this discrepancy may in part be explained by features associated with our sample. The participants in this study were former and current students in higher education, which may represent a rather resourceful and resilient group of individuals with a potentially higher threshold before gaming and high-level gaming leads to problems. Future studies should thus aim to investigate whether the observed associations exist in other populations as well. To our knowledge, the present study is the first to investigate the relationship between different levels of gaming investment and/or problems associated with gaming and different patterns of problematic alcohol use. As such, our study contributes several novel findings, and it specifies the level of gaming investment and/or problems associated with gaming that are associated (inversely or otherwise) with specific types of problematic alcohol use patterns.

The main aim in the present study was to investigate associations between gaming and problematic alcohol use. We also investigated prevalence rates of different levels of gaming investment and/or problems associated with gaming, and demographic, personality and mental health characteristics associated with different levels of gaming investment and/or problems associated with gaming. The prevalence rates should be interpreted with caution as the sample only included students and individuals who recently ended their education and as the response rate was rather low. Still, it should be noted that only 0.2% (*n* = 11) of the sample endorsed all the seven symptoms of “video game addiction” included in the GASA. Endorsing all seven symptoms is often considered as an indication of “gaming addiction” (Lemmens et al., 2009; Mentzoni et al., 2011). The current results suggest as such that substantial gaming-related problems are very rare, at least among Norwegian students. Most of our findings regarding characteristics associated with low-level and high-level gaming were in line with previous research (Ferguson et al., 2011; Kuss & Griffiths, 2012; Mentzoni et al., 2011; Peters & Malesky, 2008; Van Rooij et al., 2014; Wenzel et al., 2009; Wittek et al., 2016). Further, we found some demographic, personality, and mental health differences between the gaming groups that – to our knowledge – have not been reported previously. The observed inverse association between having children and low-level and high-level gaming, and the positive association between being a student and low-level gaming are novel findings. Individuals without children and students might have more time available to engage in gaming, compared with individuals with childcare responsibilities and former students, and this may explain why not having children and being a student were associated with gaming. The observed associations between low-level gaming and lower neuroticism, depression, and anxiety scores have – to our knowledge – not been reported before. A possible explanation for the association between low-level gaming and lower levels of neuroticism, depression and anxiety might be sex, as the low-level gamers were more likely to be men and men tend to score lower on neuroticism and report less symptoms of depression and anxiety compared to women (Lynn & Martin, 1997; World Health Organization, 2002). Another possible explanation to the inverse association between low-level gaming and neuroticism, depression and anxiety may be that gaming without palpable problems may give the gamer positive experiences of social bonding, achievement, and flow that may improve mental health (Hellström et al., 2012; Hoffman & Nadelson, 2010). A more thorough investigation of the relationship between low-level gaming, neuroticism, and mental health should be an objective for future research. The demographic, personality, and mental health differences we found between non-gamers versus low-level and high-level gamers, and the changed pattern of results when controlling for these variables on the associations between gaming and problematic alcohol use, highlight the importance of including multifarious covariates in such predictor models.

### 4.1 Limitations and strengths

The current study has several strengths, including the large sample size and the vast number of relevant covariates included in the analyses. Another asset of the present study is the differentiation between levels of gaming investment and/or problems associated with gaming and patterns of problematic alcohol use. Finally, most of the variables included in the present study were measured with standardized scales with good reliability and validity.

In terms of limitations, it should be noted that the present study is based on data from a follow-up survey, where the response rate in the original study was 39.4%. The low response rate in the original study may restrict the representativeness of the present sample. A response rate of 39.4% is, however, rather good compared to similar studies (Nedregård & Olsen, 2014; Sheehan, 2001), and the participants in our study had similar characteristics with regards to sex, age, relationship status, and alcohol use as those found in other studies among Norwegian students (Nedregård & Olsen, 2014; Statistisk sentralbyrå [Statistics Norway], 2017). Hence, it may be reasonable to assume that the current results are at least generalizable to the Norwegian student population. It is, however, possible that gaming and problematic alcohol use will relate differently to each other in other populations. Another limitation with the current study is the cross-sectional design, which precludes conclusions regarding directionality and causality. Hence, the directionality between gaming and problematic alcohol use remains unknown. Finally, some interesting measurements of gaming behaviour, that may relate differently to alcohol use (i.e. time spent gaming and type of games played) were not assessed in the present study.

## 5 Conclusions

The present study found a co-occurrence of low-level gaming and problematic alcohol use. Demographic characteristics of low-level gamers seemed to account for the co-occurrence between low-level gaming and problematic alcohol use. The inverse relationship between high-level gaming and problematic alcohol use (when adjusting for demographic, personality, and mental health variables) suggest that heavy investment in gaming may protect against excessive alcohol use and alcohol-related harm. Possible mechanisms through which high-level gaming may protect against problematic alcohol use includes high-level gamers having less available time to drink, intoxication being incompatible with gaming, and/or high-level gamers experiencing sufficiently satisfaction/escape and social bonding by gaming, hence having less need for alcohol.

Future studies should aim to investigate the relationship between gaming and alcohol use longitudinally and in other populations than students. The current results suggest that future studies should control for demographic, personality, and mental health factors when investigating the relationship between gaming and alcohol use.

## Funding

This work was funded by a PhD grant given to the first author from the University of Bergen, Norway and Bergen municipality, but we received no specific grant.

## Conflict of interest

None.

## References

[bb0005] Aarseth E., Bean A.M., Boonen H., Carras M.C., Coulson M., Das D., Van Rooij A.J. (2017). Scholars' open debate paper on the World Health Organization ICD-11 gaming disorder proposal. Journal of Behavioral Addictions.

[bb0010] American Psychiatric Association (2013). Diagnostic and statistical manual of mental disorders.

[bb0015] Andersson C., Johnsson K.O., Berglund M., Ojehagen A. (2007). Alcohol involvement in Swedish university freshmen related to gender, age, serious relationship and family history of alcohol problems. Alcohol and Alcoholism.

[bb0020] Andrews G., Murphy K., Vanchevsky M.A. (2006). Does video game playing improve executive functioning?. Frontiers in: Cognitive psychology.

[bb0025] Babor, T. F., Higgins-Biddle, J. C., Saunders, J. B., & Monteiro, M. G. (2001). The alcohol use disorders identification test: Guidelines for use in primary care. www.who.int: World Health Organization.

[bb0030] Baler R.D., Volkow N.D. (2006). Drug addiction: The neurobiology of disrupted self-control. Trends in Molecular Medicine.

[bb0035] Bergen H.A., Martin G., Roeger L., Allison S. (2005). Perceived academic performance and alcohol, tobacco and marijuana use: Longitudinal relationships in young community adolescents. Addictive Behaviors.

[bb0040] Bingham C.R., Shope J.T., Tang X.L. (2005). Drinking behavior from high school to young adulthood: Differences by college education. Alcoholism-Clinical and Experimental Research.

[bb0045] Boden J.M., Fergusson D.M. (2011). Alcohol and depression. Addiction.

[bb0050] Bohn M.J., Babor T.F., Kranzler H.R. (1995). The Alcohol Use Disorders Identification Test (AUDIT): Validation of a screening instrument for use in medical settings. Journal of Studies on Alcohol.

[bb0055] Boot W.R., Kramer A.F., Simons D.J., Fabiani M., Gratton G. (2008). The effects of video game playing on attention, memory, and executive control. Acta Psychologica.

[bb0060] Brunborg G.S., Mentzoni R.A., Frøyland L.R. (2014). Is video gaming, or video game addiction, associated with depression, academic achievement, heavy episodic drinking, or conduct problems?. Journal of Behavioral Addictions.

[bb0065] Buelow M.T., Okdie B.M., Cooper A.B. (2015). The influence of video games on executive functions in college students. Computers in Human Behavior.

[bb0070] Carras M.C., Kardefelt-Winther D. (2018). When addiction symptoms and life problems diverge: A latent class analysis of problematic gaming in a representative multinational sample of European adolescents. European Child & Adolescent Psychiatry.

[bb0075] Chen H., Cohen P., Chen S. (2010). How big is a big odds ratio? Interpreting the magnitudes of odds ratios in epidemiological studies. Communications in Statistics—Simulation and Computation.

[bb0080] Choi S.-W., Kim H., Kim G.-Y., Jeon Y., Park S., Lee J.-Y., Kim D.-J. (2014). Similarities and differences among internet gaming disorder, gambling disorder and alcohol use disorder: A focus on impulsivity and compulsivity. Journal of Behavioral Addictions.

[bb0085] Cohen J. (1988). Statistical power analysis for the behavioral sciences.

[bb0090] Connor J., Gray A., Kypri K. (2010). Drinking history, current drinking and problematic sexual experiences among university students. Australian and New Zealand Journal of Public Health.

[bb0095] Cooper M.L., Frone M.R., Russell M., Mudar P. (1995). Drinking to regulate positive and negative emotions: A motivational model of alcohol use. Journal of Personality and Social Psychology.

[bb0100] Derogatis L.R., Lipman R.S., Rickels K., Uhlenhuth E.H., Covi L. (1974). Hopkins symptom checklist (HSCL): Self-report symptom inventory. Behavioral Science.

[bb0105] DeSimone A., Murray P., Lester D. (1994). Alcohol use, self-esteem, depression, and suicidality in high school students. Adolescence.

[bb0110] Dishman R.K., Berthoud H.R., Booth F.W., Cotman C.W., Edgerton V.R., Fleshner M.R., Hillman C.H. (2006). Neurobiology of exercise. Obesity.

[bb0115] Dixit A.R., Crum R.M. (2000). Prospective study of depression and the risk of heavy alcohol use in women. American Journal of Psychiatry.

[bb0120] Donnellan M.B., Oswald F.L., Baird B.M., Lucas R.E. (2006). The Mini-IPIP scales: Tiny-yet-effective measures of the big five factors of personality. Psychological Assessment.

[bb0125] Durlak J.A. (2009). How to select, calculate, and interpret effect sizes. Journal of Pediatric Psychology.

[bb0130] Erevik E.K., Pallesen S., Vedaa Ø., Andreassen C.S., Torsheim T. (2017). Alcohol use among Norwegian students: Demographics, personality and psychological health correlates of drinking patterns. Nordic Studies on Alcohol and Drugs.

[bb0135] Ferguson C.J. (2009). An effect size primer: A guide for clinicians and researchers. Professional Psychology: Research and Practice.

[bb0140] Ferguson C.J., Coulson M., Barnett J. (2011). A meta-analysis of pathological gaming prevalence and comorbidity with mental health, academic and social problems. Journal of Psychiatric Research.

[bb0145] Giancola P.R., Zeichner A., Yarnell J.E., Dickson K.E. (1996). Relation between executive cognitive functioning and the adverse consequences of alcohol use in social drinkers. Alcoholism: Clinical and Experimental Research.

[bb0150] Grant B.F., Stinson F.S., Dawson D.A., Chou S.P., Dufour M.C., Compton W., Kaplan K. (2004). Prevalence and co-occurrence of substance use disorders and independent mood and anxiety disorders - results from the national epidemiologic survey on alcohol and related conditions. Archives of General Psychiatry.

[bb0155] Griffiths M. (2010). Online video gaming: What should educational psychologists know?. Educational Psychology in Practice.

[bb0160] Hellström C., Nilsson K.W., Leppert J., Åslund C. (2012). Influences of motives to play and time spent gaming on the negative consequences of adolescent online computer gaming. Computers in Human Behavior.

[bb0165] Hoffman B., Nadelson L. (2010). Motivational engagement and video gaming: A mixed methods study. Educational Technology Research and Development.

[bb0170] Ipsos MediaCT (2014). The 2014 essential facts about the computer and video game industry. http://www.theesa.com/wp-content/uploads/2014/10/ESA_EF_2014.pdf.

[bb0175] Jackson K.M. (2008). Heavy episodic drinking: Determining the predictive utility of five or more drinks. Psychology of Addictive Behaviors.

[bb0180] Jacobs D.F. (1986). A general theory of addictions: A new theoretical model. Journal of Gambling Behavior.

[bb0185] Karim R., Chaudhri P. (2012). Behavioral addictions: An overview. Journal of Psychoactive Drugs.

[bb0190] Kilpatrick M., Hebert E., Bartholomew J. (2005). College students' motivation for physical activity: Differentiating men's and women's motives for sport participation and exercise. Journal of American College Health.

[bb0195] Krossbakken E., Pallesen S., Mentzoni R.A., King D.L., Molde H., Finserås T.R., Torsheim T. (2018). A cross-lagged study of developmental trajectories of video game engagement, addiction, and mental health. Frontiers in Psychology.

[bb0200] Kushner M.G., Abrams K., Borchardt C. (2000). The relationship between anxiety disorders and alcohol use disorders: A review of major perspectives and findings. Clinical Psychology Review.

[bb0205] Kuss D.J., Griffiths M.D. (2012). Internet gaming addiction: A systematic review of empirical research. International Journal of Mental Health and Addiction.

[bb0210] Lemmens J.S., Valkenburg P.M., Peter J. (2009). Development and validation of a game addiction scale for adolescents. Media Psychology.

[bb0215] Lisha N.E., Sussman S. (2010). Relationship of high school and college sports participation with alcohol, tobacco, and illicit drug use: A review. Addictive Behaviors.

[bb0220] Liu M., Peng W. (2009). Cognitive and psychological predictors of the negative outcomes associated with playing MMOGs (massively multiplayer online games). Computers in Human Behavior.

[bb0225] Lynn R., Martin T. (1997). Gender differences in extraversion, neuroticism, and psychoticism in 37 nations. The Journal of Social Psychology.

[bb0230] Malouff J.M., Thorsteinsson E.B., Rooke S.E., Schutte N.S. (2007). Alcohol involvement and the five-factor model of personality: A meta-analysis. Journal of Drug Education.

[bb0235] Menagi F.S., Harrell Z.A., June L.N. (2008). Religiousness and college student alcohol use: Examining the role of social support. Journal of Religion and Health.

[bb0240] Mentzoni R.A., Brunborg G.S., Molde H., Myrseth H., Skouverøe K.J.M., Hetland J., Pallesen S. (2011). Problematic video game use: Estimated prevalence and associations with mental and physical health. Cyberpsychology, Behavior and Social Networking.

[bb0245] Nedregård T., Olsen R. (2014). Studentenes helse- og trivselsundersøkelse 2014. [Students' Health and Wellbeing Survey 2014]. http://www.google.no/url?sa=t&rct=j&q=&esrc=s&frm=1&source=web&cd=1&ved=0CB0QFjAA&url=http%3A%2F%2Fwww.vtbergen.no%2Fwp-content%2Fuploads%2F2013%2F10%2FVT0614_6214_SHoT2014.pdf&ei=w21HVZvGEIOvswG8woGQDw&usg=AFQjCNESqBK5IcWkVZUNzMxPA7ANwM2HzA&sig2=CahhInSPe7w4ZBNAUWdXiA.

[bb0250] O'Malley P.M., Johnston L.D. (2002). Epidemiology of alcohol and other drug use among American college students. Journal of Studies on Alcohol, Supplement.

[bb0255] Padilla-Walker L.M., Nelson L.J., Carroll J.S., Jensen A.C. (2010). More than a just a game: Video game and internet use during emerging adulthood. Journal of Youth and Adolescence.

[bb0260] Park C.L. (2004). Positive and negative consequences of alcohol consumption in college students. Addictive Behaviors.

[bb0265] Peters C.S., Malesky L.A. (2008). Problematic usage among highly-engaged players of massively multiplayer online role playing games. Cyberpsychology & Behavior.

[bb0270] Peterson J.B., Rothfleisch J., Zelazo P.D., Pihl R.O. (1990). Acute alcohol intoxication and cognitive functioning. Journal of Studies on Alcohol.

[bb0275] Przybylski A.K., Weinstein N., Murayama K. (2016). Internet gaming disorder: Investigating the clinical relevance of a new phenomenon. American Journal of Psychiatry.

[bb0280] Read J.P., Beattie M., Chamberlain R., Merrill J.E. (2008). Beyond the “binge” threshold: Heavy drinking patterns and their association with alcohol involvement indices in college students. Addictive Behaviors.

[bb0285] Ream G.L., Elliott L.C., Dunlap E. (2011). Playing video games while using or feeling the effects of substances: Associations with substance use problems. International Journal of Environmental Research and Public Health.

[bb0290] Rehm J., Room R., Graham K., Monteiro M., Gmel G., Sempos C.T. (2003). The relationship of average volume of alcohol consumption and patterns of drinking to burden of disease: An overview. Addiction.

[bb0295] Rosenthal J.A. (1996). Qualitative descriptors of strength of association and effect size. Journal of Social Service Research.

[bb0300] Sayette M.A., Creswell K.G., Dimoff J.D., Fairbairn C.E., Cohn J.F., Heckman B.W., Moreland R.L. (2012). Alcohol and group formation: A multimodal investigation of the effects of alcohol on emotion and social bonding. Psychological Science.

[bb0305] Sheehan K.B. (2001). E-mail survey response rates: A review. Journal of Computer-Mediated Communication.

[bb0310] Slutske W.S., Hunt-Carter E.E., Nabors-Oberg R.E., Sher K.J., Bucholz K.K., Madden P.A.F., Heath A.C. (2004). Do college students drink more than their non-college-attending peers? Evidence from a population-based longitudinal female twin study. Journal of Abnormal Psychology.

[bb0315] Statistisk sentralbyrå [Statistics Norway] (2017). Studenter i høyere utdanning. https://www.ssb.no/utdanning/statistikker/utuvh.

[bb0320] Sussman S., Black D.S. (2008). Substitute addiction: A concern for researchers and practitioners. Journal of Drug Education.

[bb0325] Van Rooij A.J., Ferguson C.J., Colder Carras M., Kardefelt-Winther D., Shi J., Aarseth E., Przybylski A.K. (2018). A weak scientific basis for gaming disorder: Let us err on the side of caution. Journal of Behavioral Addictions.

[bb0330] Van Rooij A.J., Kuss D.J., Griffiths M.D., Shorter G.W., Schoenmakers T.M., Van De Mheen D. (2014). The (co-) occurrence of problematic video gaming, substance use, and psychosocial problems in adolescents. Journal of Behavioral Addictions.

[bb0335] Vengeliene V., Bilbao A., Molander A., Spanagel R. (2008). Neuropharmacology of alcohol addiction. British Journal of Pharmacology.

[bb0340] Walther B., Morgenstern M., Hanewinkel R. (2012). Co-occurrence of addictive behaviours: Personality factors related to substance use, gambling and computer gaming. European Addiction Research.

[bb0345] Wechsler H., Dowdall G.W., Davenport A., Castillo S. (1995). Correlates of college-student binge drinking. American Journal of Public Health.

[bb0350] Wenzel H.G., Bakken I.J., Johansson A., Götestam K.G., Øren A. (2009). Excessive computer game playing among Norwegian adults: Self-reported consequences of playing and association with mental health problems. Psychological Reports.

[bb0355] Wiese J.G., Shlipak M.G., Browner W.S. (2000). The alcohol hangover. Annals of Internal Medicine.

[bb0360] Wittek C.T., Finserås T.R., Pallesen S., Mentzoni R.A., Hanss D., Griffiths M.D., Molde H. (2016). Prevalence and predictors of video game addiction: A study based on a national representative sample of gamers. International Journal of Mental Health and Addiction.

[bb0365] World Health Organization (2002). Gender and mental health. http://www.google.no/url?sa=t&rct=j&q=&esrc=s&source=web&cd=1&ved=0ahUKEwjMsIzDtKrZAhVFU1AKHXabCCQQFggtMAA&url=http%3A%2F%2Fwhqlibdoc.who.int%2Fgender%2F2002%2Fa85573.pdf&usg=AOvVaw33O3kyn8O_ofvjbKJXDmWB.

[bb0370] World Health Organization (2014). Global status report on alcohol and health, 2014. http://www.who.int/substance_abuse/publications/alcohol_2014/en/.

[bb0375] World Health Organization (2018). International classification of diseases for mortality and morbidity statistics (ICD-11). https://icd.who.int/.

